# A Novel Tool for High-Throughput Screening of Granulocyte-Specific Antibodies Using the Automated Flow Cytometric Granulocyte Immunofluorescence Test (Flow-GIFT)

**DOI:** 10.1100/tsw.2011.20

**Published:** 2011-02-03

**Authors:** Xuan Duc Nguyen, Thomas Dengler, Monika Schulz-Linkholt, Harald Klüter

**Affiliations:** ^1^ Institute of Transfusion Medicine and Immunology, Medical Faculty Mannheim, University Heidelberg, German Red-Cross Blood Service of Baden-Württemberg - Hessen, Germany; ^2^ Institute for Transfusion Medicine Baden-Baden, German Red-Cross Blood Service of Baden-Württemberg - Hessen, Germany

**Keywords:** granulocyte-associated antibodies, TRALI, Flow-GIFT, high-throughput screening

## Abstract

Transfusion-related acute lung injury (TRALI) is a severe complication related with blood transfusion. TRALI has usually been associated with antibodies against leukocytes. The flow cytometric granulocyte immunofluorescence test (Flow-GIFT) has been introduced for routine use when investigating patients and healthy blood donors. Here we describe a novel tool in the automation of the Flow-GIFT that enables a rapid screening of blood donations. We analyzed 440 sera from healthy female blood donors for the presence of granulocyte antibodies. As positive controls, 12 sera with known antibodies against anti-HNA-1a, -b, -2a; and -3a were additionally investigated. Whole-blood samples from HNA-typed donors were collected and the test cells isolated using cell sedimentation in a Ficoll density gradient. Subsequently, leukocytes were incubated with the respective serum and binding of antibodies was detected using FITC-conjugated antihuman antibody. 7-AAD was used to exclude dead cells. Pipetting steps were automated using the Biomek NXp Multichannel Automation Workstation. All samples were prepared in the 96-deep well plates and analyzed by flow cytometry. The standard granulocyte immunofluorescence test (GIFT) and granulocyte agglutination test (GAT) were also performed as reference methods. Sixteen sera were positive in the automated Flow-GIFT, while five of these sera were negative in the standard GIFT (anti—HNA 3a, n = 3; anti—HNA-1b, n = 1) and GAT (anti—HNA-2a, n = 1). The automated Flow-GIFT was able to detect all granulocyte antibodies, which could be only detected in GIFT in combination with GAT. In serial dilution tests, the automated Flow-GIFT detected the antibodies at higher dilutions than the reference methods GIFT and GAT. The Flow-GIFT proved to be feasible for automation. This novel high-throughput system allows an effective antigranulocyte antibody detection in a large donor population in order to prevent TRALI due to transfusion of blood products.

### INTRODUCTION

Granulocyte-reactive antibodies can lead to febrile transfusion reactions or severe pulmonary transfusion reactions known as transfusion-related acute lung injury (TRALI)[1,2]. Epidemiological, clinical, and experimental studies have demonstrated a close relationship between the occurrence of leukocyte-reactive antibodies in donors and TRALI in transfused recipients[3,4,5]. TRALI is caused by leukocyte antibodies specific against HLA class I and II, as well as by the human neutrophil antigen (HNA) system[6]. TRALI is a serious and, in some cases, fatal complication of blood transfusion. It is now the leading cause of transfusion-associated mortality[7,8], especially when granulocyte antibodies specific for the HNA-3a are involved[9].

The granulocyte immunfluorescence test (GIFT) and the granulocyte agglutination test (GAT) were introduced as the reference methods for the detection of granulocyte antibodies in the past[10,11]. However, the investigation of a large number of blood donor samples using the standard GIFT and GAT proved to be difficult to perform due to the time-consuming process needed and the large number of test cells required. The combination of both methods is at present necessary to detect all relevant antibodies to HNA, particularly HNA-3a antibodies, which can reliably detected only by GAT[12,13]. Recently, we established and introduced a novel method known as the Flow-GIFT (flow cytometric granulocyte immunofluorescence test) for the detection of granulocyte-associated antibodies[14].

In the present study, we further developed the Flow-GIFT method in automation for the rapid detection of granulocyte antibodies in order to screen large amounts of samples.

### MATERIALS AND METHODS

#### Blood Samples

We analyzed 440 sera of healthy female blood donors for the presence of granulocyte-reactive antibodies. Additionally, 12 sera with known granulocyte-specific antibodies were tested. Each sample was labeled with a barcode. Granulocyte test cells were obtained from freshly drawn $Na_2$EDTA whole blood of healthy blood donors. The donors were previously genotyped for HNA-1, -3, -4, and -5 using polymerase chain reaction with sequence-specific primers (PCR-SSP). The HNA-phenotype (HNA-1, -2 and -3) was additionally determined using the properties of the typing sera. Each donor of test cells had been analyzed multiple times (at minimum ten times) for the expression of the TRALI-relevant antigens (HNA-1a, -1b, -2a, and -3a) by serological testing. Furthermore, each donor involved in the current investigation was tested with positive control sera containing anti–HNA-1a, -1b, -2a, and -3a during the test sequence. The donors were homozygous for HNA-1a or -1b, and positive for HNA-2a, -3a, -4a, and -5a.

Sera from healthy blood donors without leukocyte-specific antibodies were used as normal controls. For each run, six sera containing anti–HNA-1a (n = 1), -1b (n = 1), -2a (n = 2), and -3a (n = 2) were used as positive controls. All tested sera were investigated in the conventional GAT and GIFT, as well as in the automated Flow-GIFT.

#### GIFT and GAT, Differentiation of Granulocyte-Specific Antibodies and Detection of HLA Antibodies

GIFT and GAT as standard assays were performed as described[10,11,14]. The differentiation of granulocyte-specific antibodies in GIFT and GAT was performed by the monoclonal antibody–specific immobilization of granulocyte antigens assay (MAIGA)[15] with MoAb: CD16 clone 3G8, CD16 clone DJ130c, CD18 clone 7E4, CD11a clone 25.3, CD11b clone Bear-1 (all from Immunotech, Marseilles, France) and CD177 clone MEM 166 (Becton Dickinson, Heidelberg, Germany). For detection of HLA antibodies, the enzyme-linked immunosorbent assay (AB Screen ELISA; Biotest, Dreieich, Germany) was used.

### Flow-GIFT

The isolation of test granulocytes was performed according to our protocol described[14]. Briefly, 9 ml EDTA anticoagulated blood of two healthy blood donors was added to 15 ml Ficoll density gradient (Ficoll-Paque Plus, GE Healthcare, Uppsala, Sweden) without centrifugation. After lysis of remaining erythrocytes by adding 2 ml VersaLyse™ (Beckman Coulter, Krefeld, Germany), the cells were washed and resuspended in PBS. Testing sera (50 µL/well) were prepared in a 96-deep well plate. Then, 50 µl of resuspended cells (ranging between $1 \times 10^5$ and $2.5 \times 10^5$ cells) from the first test cell donor were added in each well and incubated for 30 min at 37°C, followed by two washing steps and incubation with FITC-labeled rabbit $F(ab')_2$-antihuman IgG (Dako, Hamburg, Germany). In order to exclude dead cells, 7-AAD (Beckman Coulter, Krefeld, Germany) was added. After 30-min. incubation at room temperature, one washing step, and resuspension of 300 µL of fixative solution (Beckman Coulter), cell-bound fluorescence was detected by flow cytometric analysis. The same procedure was performed with the second test cell donor. All pipetting steps were performed using the Biomek NX$^p$ Multichannel (96 channels) Automation Workstation due to the program calculated for the Flow-GIFT procedure (Fig. 1). Only centrifugation of samples during washing steps and incubation at 37°C were performed manually. The investigator transferred the plate into the centrifuge or incubator for the necessary time and put it back into the work station.

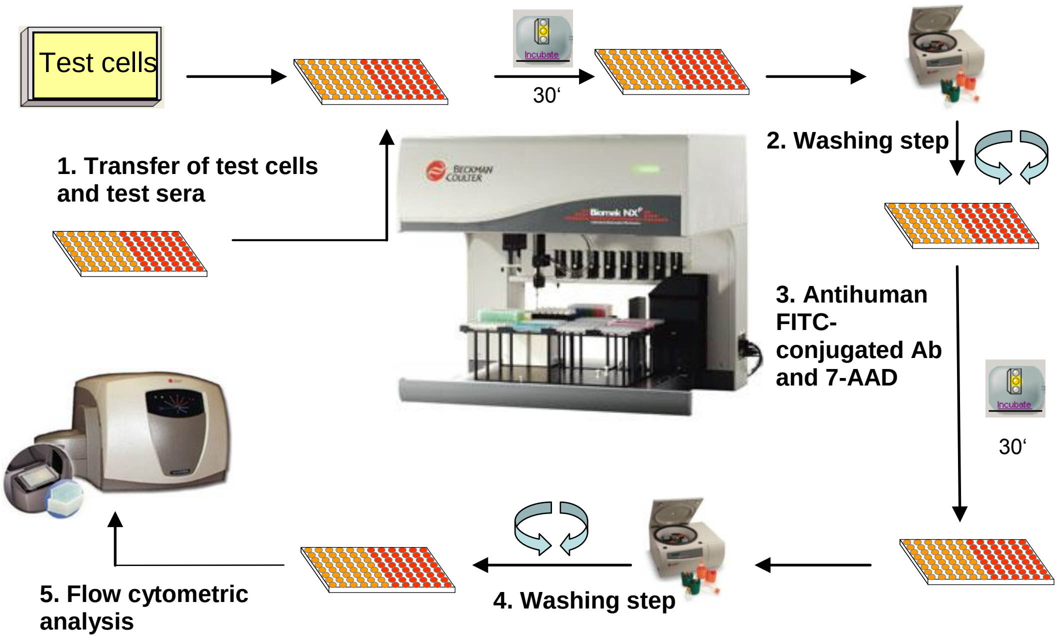

**FIGURE 1**. The principle of the automated Flow-GIFT. Test sera were prepared in a 96-deep well plate. The first column includes negative controls (positions 1 and 2) and positive controls (positions 3–8). Columns 2–12 include testing sera. Test cells were manually isolated and resuspended in a box. The Biomek NX$^p$ Multichannel Automation Workstation transfers test cells to each serum cavity. After incubation at 37°C, the samples were washed twice. Antihuman IgG-FITC and 7-AAD were added and incubated at room temperature, followed by one washing step and resuspension in fixative solution. The samples are then ready for flow cytometric analysis on cell-bound fluorescence. The same procedure was performed with the second test cell donor. Only centrifugation of samples during washing steps and incubation at 37°C were performed manually. The investigator transferred the plate into the centrifuge or incubator for the necessary time and put it back into the work station.

All flow cytometric analyses were performed on the FC 500 machine (Beckman-Coulter). The median fluorescence intensity (MFI) was given as value (Fig. 2). The cut-off was calculated as ratio of MFI of the test serum and control serum. The serum was defined as positive if the ratio value resulted >2.

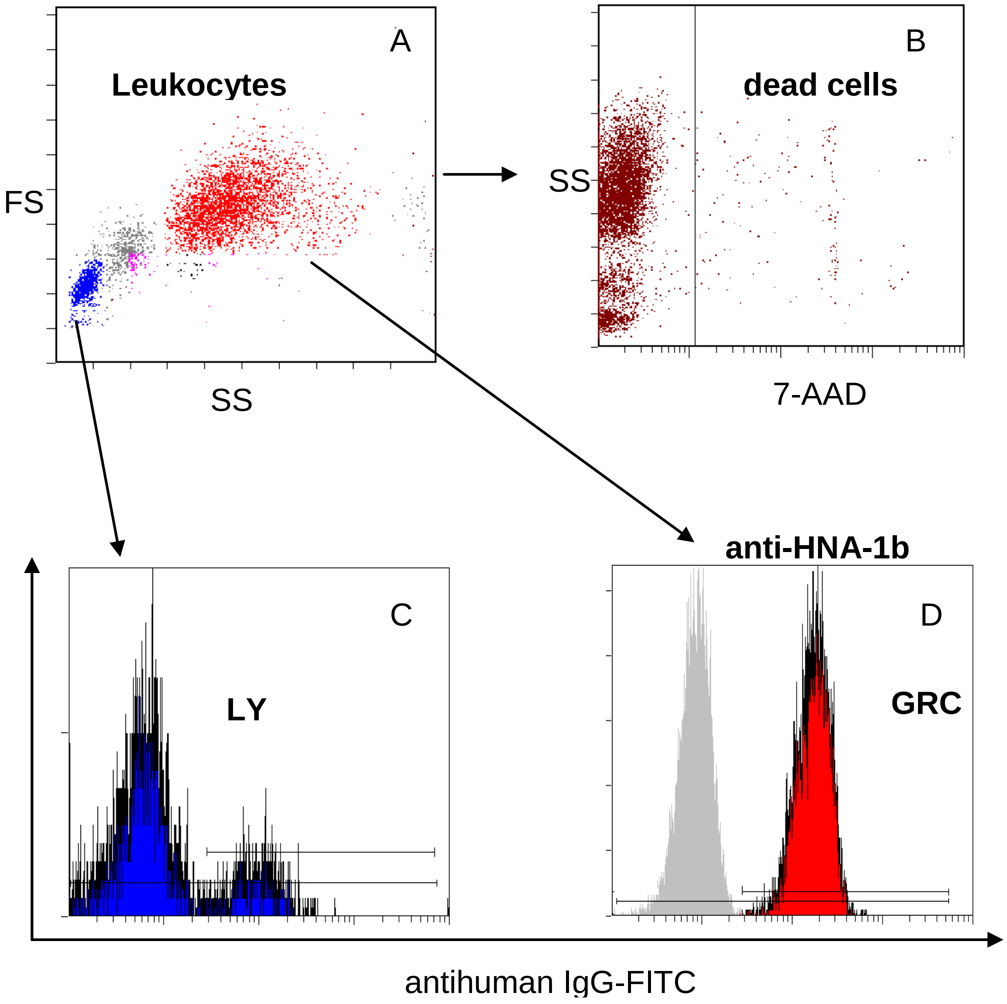

**FIGURE 2.** An example of flow cytometric analysis of a serum containing granulocyte-specific IgG anti–HNA-1b. Granulocytes and lymphocytes were separately investigated on cell-bound fluorescence in each histogram composed by the first fluorescence channel (FITC) and count. This antibody bound only on granulocytes due to the expression of HNA-1b on granulocytes (GRC) and absence of this antigen on lymphocytes (LY). This anti–HNA-1b does not show cytotoxic effect (7-AAD negative). The shadow indicates negative control.

### Titration of Sera Containing Specific Granulocyte-Binding Antibodies and Accuracy Testing of the Automated Flow-GIFT

Sera containing antibodies against HNA-1a, -1b, and -3a were serially diluted with AB serum. In order to test the accuracy of the automated Flow-GIFT, a negative control serum and a serum containing anti–HNA-1b were prepared in each well of two 96-deep well plates and analyzed, respectively.

### Statistical Analysis and Calculations

Means, standard deviations, and coefficients of variation were calculated as indicated. Differences between data were considered statistically significant at $p < 0.05$.

## RESULTS

### Testing of Sera

Sera of 440 healthy female blood donors were tested in the automated Flow-GIFT vs. standard GIFT and GAT. Additionally, 12 sera with historically positive test results for granulocyte-specific antibodies against anti–HNA-1a (n = 1), -1b (n = 3), -2a (n = 1), and -3a (n = 7) were tested in an interlaboratory analysis and confirmed in the automated Flow-GIFT. The specificity of antibodies was confirmed by MAIGA. All 12 sera were positive in the automated Flow-GIFT and GAT, but three sera containing anti–HNA-3a were not detected in standard GIFT. Out of the sera from 440 female blood donors, 436 sera were negative in both assays (Table 1). Four sera containing anti–HNA-1b (n = 2), -2a (n = 1), and -3a (n = 1) were positive in the automated Flow-GIFT. Out of these sera, one serum containing anti–HNA-1b and one serum containing anti–HNA-2a were negatively tested in standard GIFT and GAT, respectively.

**TABLE 1**
**Investigation of 452 Sera**

| Sera | Automated Flow-GIFT | | Standard GIFT | | Standard GAT | |
|---|---|---|---|---|---|---|
| | + | - | + | - | + | - |
| 16 Positive sera | 16 | — | 12 | 4 | 15 | 1 |
| 436 Negative sera | 0 | 436 | 0 | 436 | 0 | 436 |

The amounts of sera with positive- and negative-tested granulocyte-binding IgG in automated Flow-GIFT vs. standard GIFT and standard GAT.

Twenty-nine sera were positively tested for HLA-class I and II antibodies. Ten sera contained anti–HLA-class I, six sera HLA-class II, one and 13 sera both class I and II antibodies.

### Sensitivity and Accuracy of the Automated Flow-GIFT

To determine the sensitivity of the automated Flow-GIFT, three sera containing known specific granulocyte-binding antibodies (anti–HNA-1a, -1b, and -3a) were diluted with AB serum and investigated in the automated Flow-GIFT, standard GIFT, and standard GAT. Table 2 depicts the serial dilution of the sera. All measured values of the serial dilutions decreased in direct proportion to the concentration used. In all sera, the automated Flow-GIFT detected granulocyte-specific antibodies at higher dilutions than the standard GIFT and/or GAT.

In order to investigate the accuracy of the automated Flow-GIFT, serial testing of one control serum and one serum containing anti–HNA-1b was performed in two 96-deep well plates. Then, 96 measured values of each plate were calculated. All cavities with control serum and serum with anti–HNA-1b were negative and positive, respectively. The results of both sera showed a high significant difference with $p < 0.0001$. The mean MFI values of control serum and serum containing anti–HNA-1b were at $0.81 \pm 0.07$

**TABLE 2**
**Serial Dilutions of Sera Containing Specific Human IgG Against HNA-1a, -1b, -and -3a**

| Specific Antibodies | Titer | | |
|---|---|---|---|
| | Automated Flow-GIFT | Standard GIFT | Standard GAT |
| Anti–HNA-1a | 64 | 2 | 2 |
| Ant–HNA-1b | 64 | 4 | 2 |
| Anti–HNA-3a | 64 | — | 16 |

The titers in automated Flow-GIFT, standard GIFT, and standard GAT are depicted. The automated Flow-GIFT proves to be more sensitive than standard GIFT and/or GAT.

(0.64–0.95), with an average standard deviation (coefficient of variation) of 9.2%, and at 19.1 ± 1.83 (14.2–22.4), with a CV of 9.61%, respectively (Fig. 3A, B).

### DISCUSSION

The essential restrictions of the current methods used to investigate granulocyte-associated antibodies in a large amount of samples in parallel lie in the high amount of test cells required and the missing automation. Recently, we developed a novel assay, Flow-GIFT, for rapid detection of granulocyte-associated antibodies using a novel isolation technique using Ficoll density gradient without centrifugation and flow cytometric analysis[14]. This study is a further step to develop an automation process and thus is the first performance of a novel technology for high-throughput investigation on granulocyte-associated antibodies. For the investigation of 176 samples without controls, a time span of 8 h is required. For the whole investigation, 30 ml of EDTA-blood are needed, and one technician is involved.

In comparison to the manual Flow-GIFT, more test cell resuspension (1 ml) is required in order to cover the dead volume in automation.

Our data indicate that the automated Flow-GIFT was superior for detecting granulocyte-associated antibodies, both in undiluted as well as in our dilution experiments in comparison to standard GIFT and GAT. There was a large analogy for the detection of antibodies between the automated Flow-GIFT vs. standard GIFT in combination with GAT. This result indicates that the automated Flow-GIFT alone without the use of agglutination assay is able to detect granulocyte-associated antibodies in comparison to the standard assays. Due to the use of the entire leukocyte fractions as test cells resulting in simultaneous evaluation of cell-bound fluorescence on granulocytes and lymphocytes, the binding antibody can be specified in many cases without the use of differentiation assay, e.g., MAIGA, which is lab and time consuming.

In order to investigate the accuracy of the automated Flow-GIFT, a serial testing of a control serum and a serum containing an anti–HNA-1b was performed. The low CV value supports the accuracy of the automated Flow-GIFT.

In this study, HLA antibodies were not recognized by the automated Flow-GIFT as well as standard GIFT and GAT. A likely explanation for this is that for ELISA, a pool of 300 donors was used and this pool was tested to react with the most common HLA antibodies. By the automated Flow-GIFT as well as standard GIFT and GAT, in contrast, single donors were tested and we therefore may have missed some antibodies directed against HLA antigens that were not included.

In conclusion, the automated Flow-GIFT allows rapid and simple detection of granulocyte antibodies, while less donor test cells are needed than for the current methods. This high-throughput method paves the way for the screening of granulocyte antibodies in large donor populations. Thus, antibodies against new antigens may be detected. This will help us to detect granulocyte antibodies in blood donors and help to reduce TRALI.

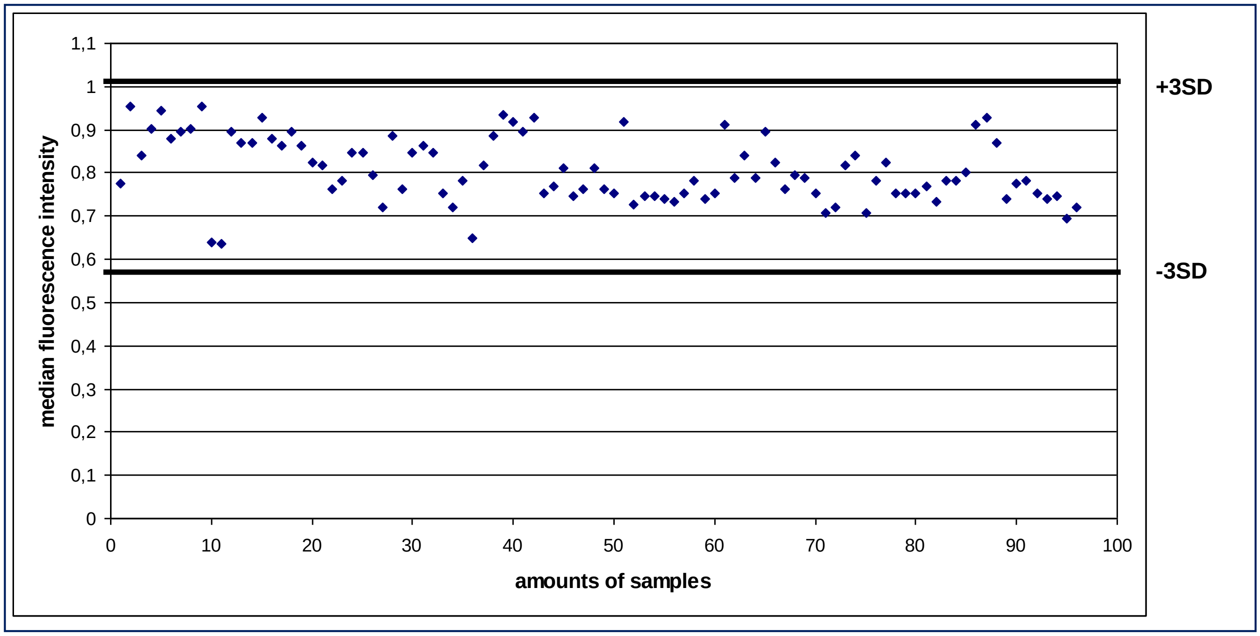

A

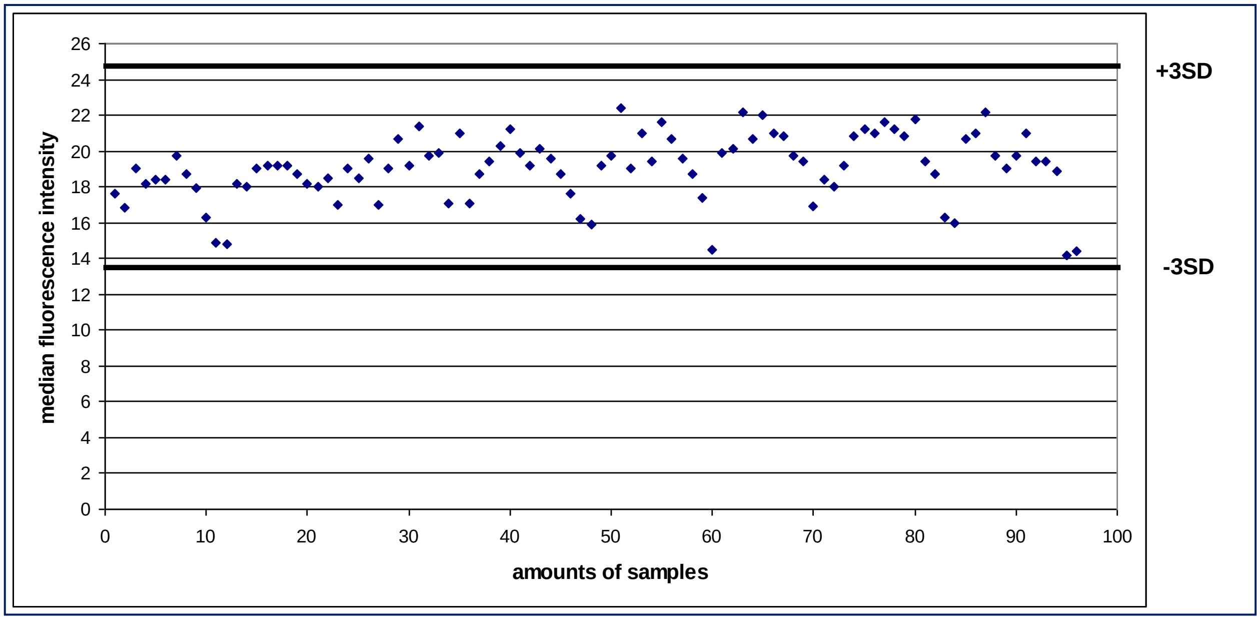

B

**FIGURE 3.** The accuracy of the automated Flow-GIFT was analyzed. Control serum (A) and a serum containing anti–HNA-1b (B) were prepared in each cavity of two 96-deep well plates and investigated. Then, 96 measured values (MFI) of each plate were calculated. All cavities with control serum and serum with anti–HNA-1b were negative and positive, respectively. The MFI values show a normal distribution (Kolmogorov Smirnov test) and are located within the range of mean ± 3 SD.
